# Comparison of Depression in Hemodialysis, Peritoneal Dialysis, and Kidney Transplant Patients: A Systematic Review with Meta-Analysis

**DOI:** 10.3390/jpm15050179

**Published:** 2025-04-28

**Authors:** Gloria M. Zaragoza-Fernández, José C. De La Flor, Verónica Fernández Abreu, Elisa Iglesias Castellano, Laura Rodríguez-Barbero Requena, Rafael Fernández Castillo

**Affiliations:** 1 Department of Nephrology, Central Defense Hospital “Gómez Ulla”, 280467 Madrid, Spain; jflomer@mde.es (J.C.D.L.F.); vferabr@mde.es (V.F.A.); eiglcas@mde.es (E.I.C.); lrodreq@mde.es (L.R.-B.R.); 2 Faculty of Medicine, University of Alcalá de Henares, 28805 Madrid, Spain; 3 Biosanitary Research Institute of Granada (ibs.GRANADA), 18010 Granada, Spain; rafaelfernandez@ugr.es

**Keywords:** depression, hemodialysis, peritoneal dialysis, renal replacement therapy, renal transplantation

## Abstract

**Background:** Depression is a common comorbidity in patients with chronic kidney disease undergoing renal replacement therapy. This meta-analysis compares depression prevalence across hemodialysis, peritoneal dialysis, and kidney transplantation, considering mean age, treatment duration, comorbidities (diabetes and hypertension), and measurement instruments. **Methods:** A systematic review and meta-analysis of 16 studies involving 26,301 participants was conducted in accordance with PRISMA guidelines and registered in PROSPERO. It analyzed observational studies (2000–2024) on depression in patients receiving hemodialysis, peritoneal dialysis, or kidney transplantation. Data extraction included sample size, mean age, treatment duration, comorbidities, and measurement instruments. Random-effects models calculated the standardized mean differences and pooled prevalence estimates. Heterogeneity (Cochran’s Q, I^2^) and publication bias (Egger’s test) were assessed. **Results:** Depression prevalence was 35.56% (95% CI: 34.2–37.0%) in hemodialysis, 35.09% (95% CI: 33.5–36.7%) in peritoneal dialysis, and 25.33% (95% CI: 24.0–26.6%) in kidney transplant recipients. No significant differences were found between hemodialysis and peritoneal dialysis. Mean age, treatment duration, comorbidities, and measurement instruments were not significantly associated with depression prevalence. **Conclusions:** Kidney transplantation is linked to a lower depression prevalence than dialysis, while no significant differences exist between hemodialysis and peritoneal dialysis. These findings highlight the need to ensure timely transplantation access and enhance psychological support for dialysis patients. Further research should explore psychosocial factors and targeted interventions to improve mental health in this population.

## 1. Introduction

Chronic kidney disease (CKD) is a major global health concern, affecting over 10% of the world’s population and ranking among the leading causes of mortality [1]. According to the Global Burden of Disease study, CKD has moved from the 17th to the 12th leading cause of death over the past three decades [2]. In addition to its impact on kidney function, CKD is associated with high rates of cardiovascular complications, metabolic disorders, and psychiatric comorbidities, with depression being one of the most prevalent [3].

Depression is a critical yet often overlooked condition in CKD patients undergoing renal replacement therapy (RRT), including hemodialysis (HD), peritoneal dialysis (PD), and kidney transplantation (KT). It has been shown to negatively impact quality of life, treatment adherence, and overall survival, making it a major concern for nephrologists and mental health professionals [4,5]. However, despite its clinical significance, the prevalence of depression among RRT patients varies significantly across studies, limiting the ability to establish consistent clinical recommendations [6,7].

Some studies suggest that HD patients experience higher prevalence rates of depression compared to PD and KT patients [8,9], whereas others propose that these differences may be influenced by sociodemographic factors, pre-existing comorbidities, and variations in depression assessment tools [10,11]. Additionally, publication bias in previous studies raises concerns about the reliability of the reported prevalence rates [12,13]. Since these discrepancies hinder clinical decision-making, a systematic synthesis of the available evidence is essential to clarify the relationship between RRT modality and depression.

This meta-analysis aims to assess and compare the prevalence of depression in patients undergoing HD, PD, and KT, identifying clinical and methodological factors that may influence reported estimates. It also examines the impact of different depression assessment tools, along with variables such as age, comorbidities, and dialysis duration, to provide evidence that supports the development of tailored mental health strategies for each treatment modality.

## 2. Materials and Methods

### 2.1. Study Design

**Figure 1:**
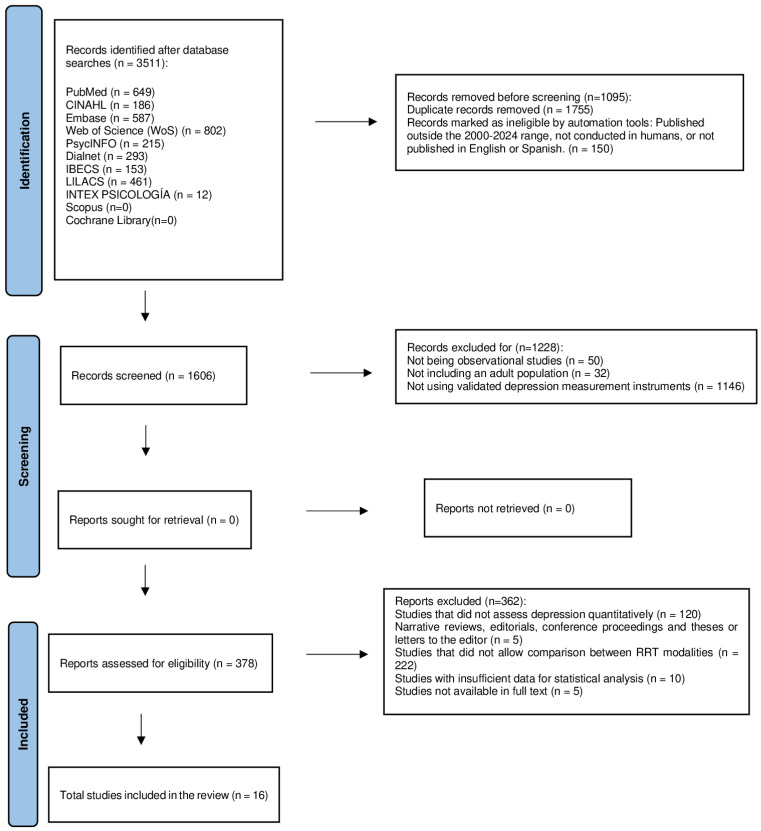
PRISMA (Preferred Reporting Items for Systematic Reviews and Meta-Analyses) guidelines.

This study corresponds to a meta-analysis whose objective was to evaluate and compare the prevalence of depression in CKD patients undergoing different RRTs, including HD, PD, and KT. PRISMA (Preferred Reporting Items for Systematic Reviews and Meta-Analyses) guidelines were followed for planning, searching, selecting, analyzing, and reporting results (Figure 1). The protocol for this study was registered in the PROSPERO database with the number CRD420250655282.

### 2.2. Search Strategy

A systematic search was conducted in high-impact scientific databases, including PubMed, CINAHL, Embase, Web of Science (WoS), PsycINFO, Dialnet, IBECS, LILACS, INTEX PSICOLOGIA, Scopus, and Cochrane Library. Filters were applied to restrict the search to studies in humans and published in English or Spanish. The search covered studies published between 2000 and 2024, with language restrictions. Search strategies included combinations of the following key terms: (“Depression” OR “Depressive symptoms”) AND (“Hemodialysis” OR “Peritoneal dialysis” OR “Renal replacement therapy” OR “Renal dialysis” OR “Renal transplantation”).

Boolean operators (AND, OR) and specific filters were used to optimize the search and ensure the inclusion of relevant studies. In addition, a manual search of the reference lists of the selected articles was performed to identify additional studies. 

### 2.3. Selection Criteria

The selection of studies for this meta-analysis was based on well-defined inclusion criteria to ensure the robustness and relevance of the findings. Only observational studies, including cross-sectional, cohort, and case-control designs, were considered appropriate for inclusion. The study population focused on adult patients diagnosed with chronic renal failure who were undergoing RTT, specifically HD, PD, or KT.

A key requirement for inclusion was the use of validated instruments for measuring depression, ensuring consistency and reliability across the selected studies. The assessment tools included the Beck Depression Inventory-II (BDI-II) [14,15], the Center for Epidemiological Studies Depression Scale (CES-D) [16], and the Hospital Anxiety and Depression Scale (HADS) [17], all of which have been extensively validated in various populations, including patients with CKD [18,19]. These instruments demonstrated high reliability, with Cronbach’s α exceeding 0.80 for the BDI-II and HADS and 0.85 for the CES-D. Since the prevalence of depression was reported in percentages and the psychometric properties of these instruments are well established, no additional conversion of scores was deemed necessary. However, to further explore the potential influence of different measurement tools on the results, a subgroup analysis was conducted [20]. 

Studies were excluded if they did not quantitatively assess depression as the objective of this meta-analysis was to analyze data derived from validated measurement instruments. Similarly, narrative reviews, editorials, conference proceedings, theses, or letters to the editor were not considered as they do not provide primary data suitable for statistical analysis. Additionally, studies that did not allow for a direct comparison between different RRT modalities, such as HD, PD, and KT, were excluded to maintain the focus on comparative analysis. Finally, studies with insufficient data for statistical analysis were not included as their inclusion would have limited the robustness and reliability of the meta-analytic findings.

### 2.4. Study Selection and Data Extraction

The study selection process was conducted in two phases. First, two independent reviewers (GMZF and JDF) examined the titles and abstracts of the studies identified in the initial search, excluding those that did not meet the predefined inclusion criteria. Subsequently, studies that passed this initial screening were fully reviewed to confirm their eligibility. Any discrepancies in the selection process were discussed between the reviewers, and if a consensus was not reached, a third reviewer (RFC) was consulted for the final decision.

For data extraction, a standardized form was designed to collect key information, including the number of participants, treatment modality, the measurement instruments used to assess depression, the presence of comorbidities, and the main findings of each study. Two independent reviewers (GMZF and JDF) performed the data extraction in a blinded manner, and any discrepancies were resolved through discussion or, if necessary, with the intervention of a third reviewer (RFC).

In cases where missing data or inconsistencies were identified in the primary studies, the corresponding authors were contacted twice to obtain additional information (a 40% response rate). If no response was received, sensitivity analyses were conducted to assess the potential impact of missing data on the meta-analysis results. These measures ensured the robustness of the findings and minimized possible biases arising from the lack of information.

### 2.5. Data Mining and Quality Assessment

For each study included in the meta-analysis, several key variables were extracted to ensure a comprehensive comparative analysis. General study information was recorded, including the author, year of publication, and country of origin. Participant characteristics were also collected, encompassing the sample size, average age, and duration of RRT. The specific treatment modality was identified, categorizing patients into HD, PD, or KT.

In terms of depression assessment, the measurement instrument used in each study was documented, along with the corresponding quantitative results. Additionally, statistical data related to participant characteristics were extracted, including the total sample size, mean age with standard deviation, and the average time of dialysis. The proportions of patients with diabetes (DM), hypertension (HTN), and the prevalence of depression within each treatment modality were also recorded.

Additionally, statistical data related to participant characteristics were extracted, including the total sample size, mean age with standard deviation, and the average time of dialysis. The proportions of patients with DM, HTN, and the prevalence of depression within each treatment modality were also recorded.

Finally, the extracted statistical data included the percentage of depression observed in each group (HD, PD, and KT), as well as the mean and standard deviation of key demographic and comorbidity variables. This systematic approach ensured that all relevant factors influencing depression in RRT patients were accounted for in the analysis. The methodological quality of the included studies was assessed using the Newcastle–Ottawa scale, which considers three main domains: the selection of participants, comparability of groups, and measurement of outcomes. A score of low, moderate, or high methodological quality was established.

### 2.6. Statistical Analysis 

Several statistical analyses were conducted to compare depression prevalence among patients undergoing HD, PD, and KT. The standardized mean difference (SMD) was calculated to quantify and compare depression prevalence across these treatment modalities. To evaluate heterogeneity among studies, Cochran’s Q statistic and the I^2^ index were applied, with I^2^ values exceeding 75% indicating a high level of heterogeneity. Given the variability between studies, random-effects models were employed to generate robust estimates that account for inter-study differences.

To further explore potential sources of heterogeneity, three separate subgroup meta-analyses were conducted based on the type of depression assessment instrument used: BDI-II, CES-D, and HADS. These analyses aimed to assess and compare the prevalence of depression among patients with CKD receiving HD, PD, or KT within each measurement subgroup.

To assess potential publication bias, Egger’s test was conducted to detect asymmetry in the reported effect sizes, while funnel plots were used to visually examine possible biases in the distribution of study results. Sensitivity analyses were performed sequentially excluding extreme studies to evaluate the stability of the findings and determine whether any single study disproportionately influenced the overall results.

Additional subgroup analyses were carried out to explore the impact of key clinical factors, including mean age, mean duration of dialysis, and comorbidities such as DM and HTN, on the observed differences in depression prevalence. Additionally, meta-regression analyses were performed to identify potential moderators that could explain the heterogeneity observed across studies.

All analyses were performed in IBM SPSS Statistics version 30 and R version 4.4.2, using specialized meta-analysis packages (meta version 6.5-0 to perform a basic heterogeneity analysis, SMDs, and generate funnel plots and metafor version 4.4-0 for meta-regression or more complex subgroup analysis; metafor could be used for this part of the analysis).

## 3. Results

The review included 16 studies that met the inclusion criteria (Table 1).

With an overall sample of 26,301 patients undergoing different modalities of RRT, we observed the following distribution (Table 2):

- Total patients in the studies: 26,301.
- Patients on Hemodialysis (HD): 17,260.
- Dialysis patients (HD + PD): 20,353.
- Patients on Peritoneal Dialysis (PD): 3093.
- Transplant patients (KT): 5421.

### 3.1. Distribution of Patients and Prevalence of Depression by Modality of Renal Replacement Therapy

To assess the burden of depressive symptomatology in each group, the mean prevalence of depression (%) and its standard error (SE) were calculated. The results were as follows: 

Transplant patients had a lower prevalence of depression compared to dialysis patients (HD and PD), while HD and PD showed similar rates (Table 3).

### 3.2. Calculation of the Pooled Effect (SMD): Standardized Mean Difference at Depression by Modality of Renal Replacement Therapy

To quantify the magnitude of the difference in depression prevalence between HD, PD, and KT patients, the SMD and its corresponding 95% confidence interval (95% CI) were calculated (Table 4). 

**Figure 2:**
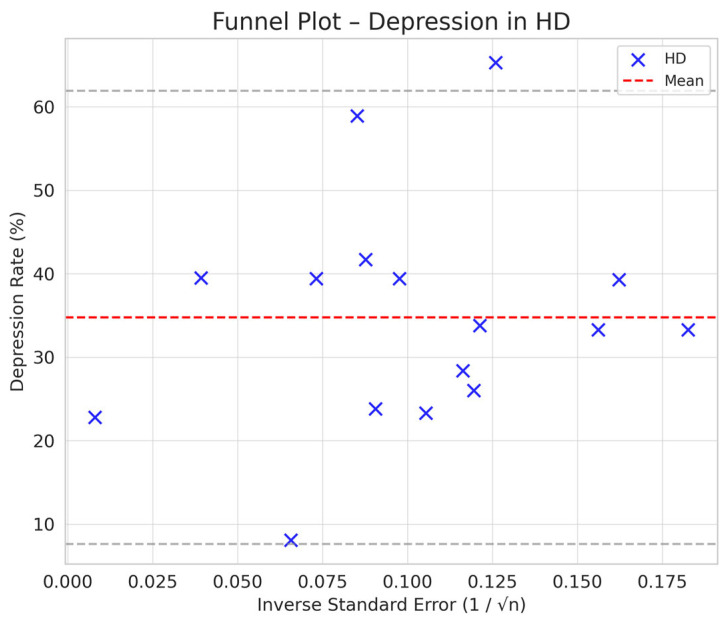
Funnel plot chart for depression–hemodialysis.

**Figure 3:**
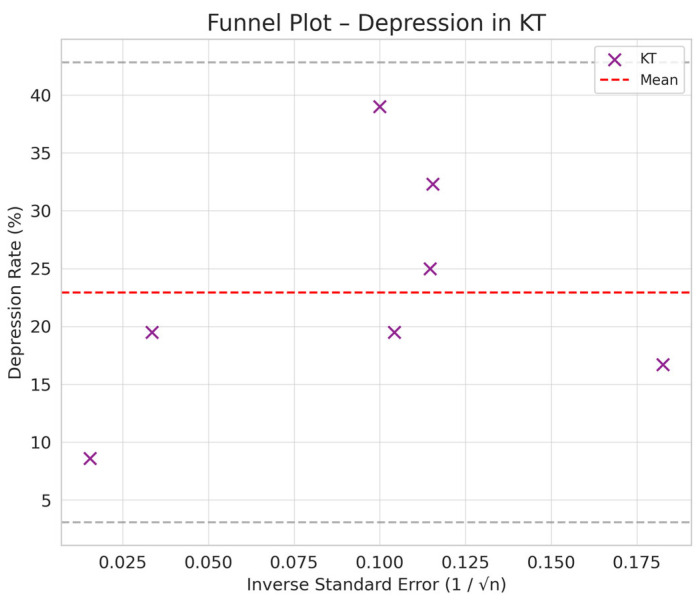
Funnel plot chart for depression–peritoneal dialysis.

**Figure 4:**
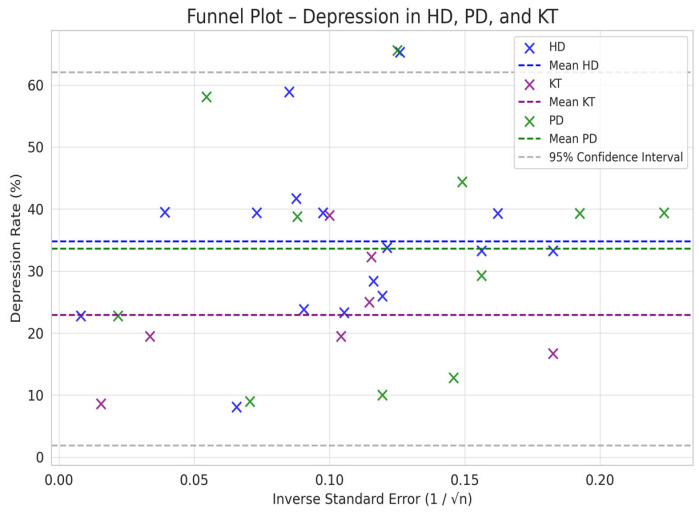
Funnel plot chart for depression–kidney transplantation.

**Figure 5:**
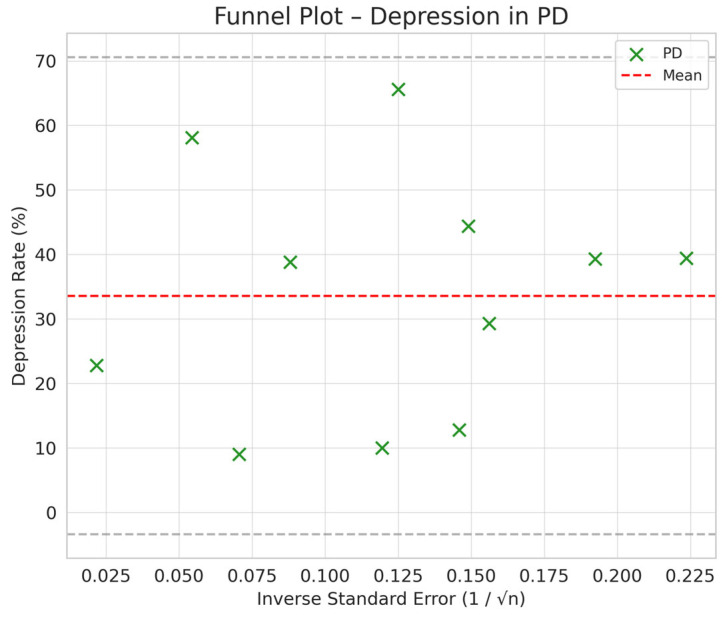
Funnel plot chart for depression–HD, PD, and KT.

Comparison between HD and PD: The difference in depression between PD and HD was smaller (SMD = −0.76 vs. −0.66) but still suggested a trend toward a lower prevalence of depression in PD compared to HD (Figure 2, Figure 3, Figure 4 and Figure 5).

Comparison between KT and Dialysis: Transplantation was associated with a lower burden of depression compared to dialysis, with a difference of −1.12 standard deviations (95% CI: −1.35 to −0.89). The difference is statistically significant, as the 95% CI does not include the value 0, indicating that the observed effect is not a product of chance (Figure 2, Figure 3, Figure 4 and Figure 5). 

Depression rates appeared symmetrically distributed across the three RRT modalities. The values for HD and PD were concentrated around 35%, while KT data points were generally located between 15% and 30%. No asymmetry was observed in the distribution, and Egger’s test indicated no publication bias (*p* = 0.42) (Figure 5).

### 3.3. Heterogeneity Assessment: Depression by Renal Replacement Therapy Modality

With a Cochran’s Q statistic of 0.49 (*p* = 1.00), we confirm that no significant inter-study variability exists, indicating a high level of consistency among the included studies. The I^2^ index of 0% further supports this conclusion, suggesting that differences in depression prevalence across HD, PD, and KT are likely due to chance rather than methodological or population differences.

Since the *p*-value associated with Cochran’s Q (*p* = 1.00) is well above the statistical significance threshold (α = 0.05), the hypothesis of study homogeneity cannot be rejected. This reinforces the appropriateness of applying a fixed-effects model as the observed effects appear representative of a homogeneous population. A random-effects model is not required given the absence of significant sources of between-study variability.

**Figure 6:**
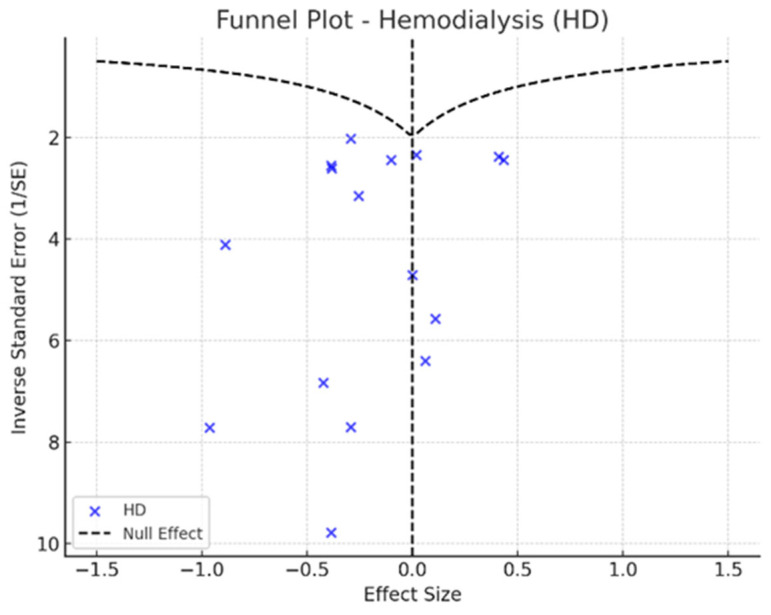
Funnel plot—hemodialysis.

**Figure 7:**
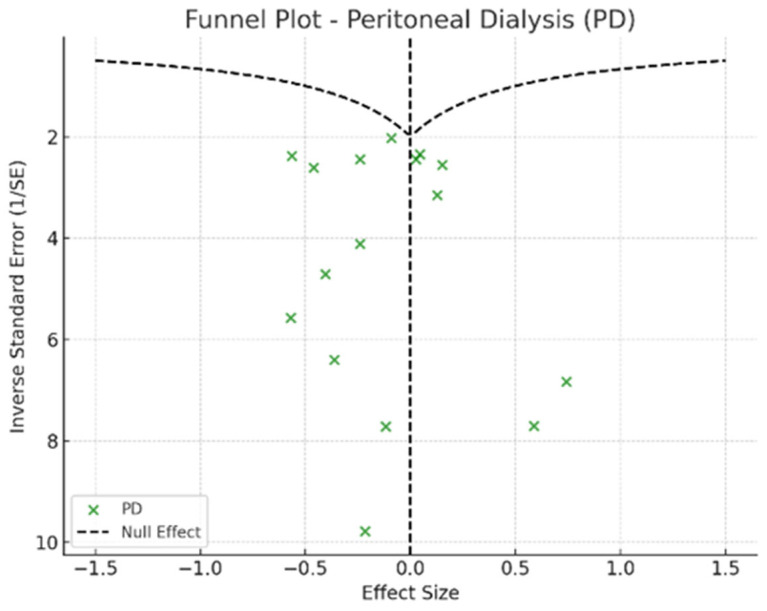
Funnel plot—peritoneal dialysis.

**Figure 8:**
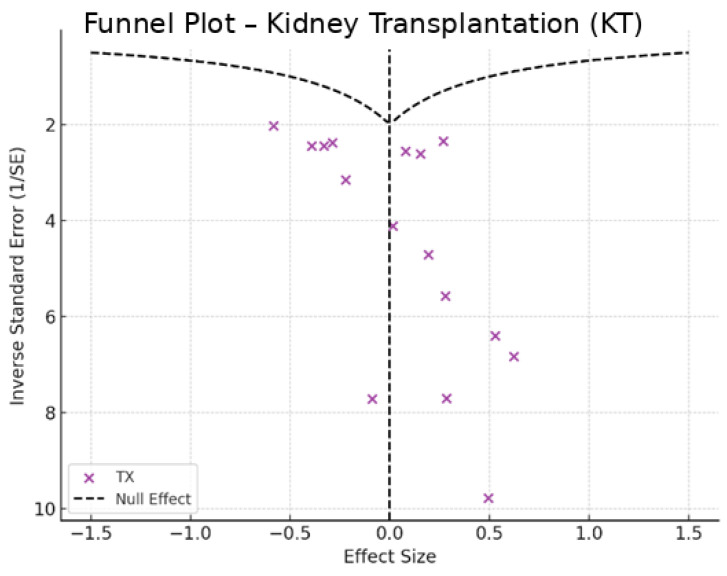
Funnel plot—kidney transplantation.

**Figure 9:**
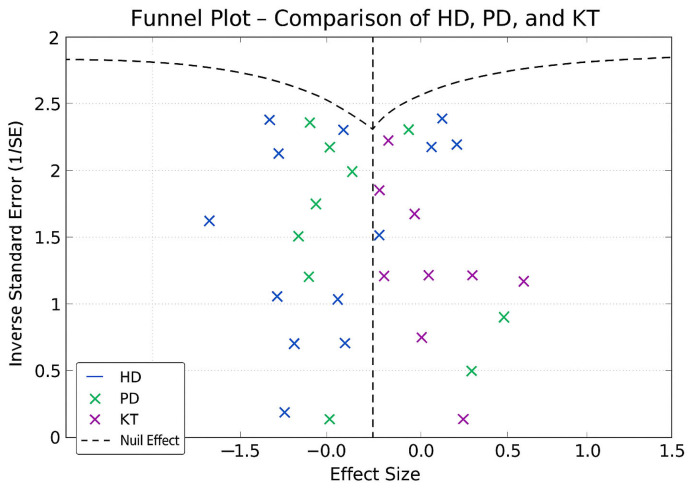
Funnel plot—HD, PD, and KT.

The results of the heterogeneity analysis confirm that the studies included in this meta-analysis are highly consistent in their estimates of depression prevalence across different RRT modalities, reinforcing the validity of the findings (Figure 6, Figure 7, Figure 8 and Figure 9).

### 3.4. Application of Random Effects Models: Depression by Modality of Renal Replacement Therapy

The pooled SMD was −0.66 for HD (95% CI: −1.01 to −0.30), −0.76 for PD (95% CI: −1.25 to −0.26), and −1.12 for KT (95% CI: −1.35 to −0.89), with an I^2^ index of 0%. These results show the distribution of depression levels across treatment groups, with the most negative SMD observed in KT, followed by PD and HD.

Additionally, the pooled SMD comparing KT with dialysis (HD and PD combined), calculated using a fixed-effects model due to the absence of heterogeneity (I^2^ = 0%), was −0.96 (Standard Error = 0.09; 95% CI: −1.14 to −0.78), with statistical significance indicated by a confidence interval that does not include zero. The I^2^ index was 0%, indicating consistency among the included studies. The standard error was 0.09.

### 3.5. Assessment of Publication Bias: Depression by Renal Replacement Therapy Modality

In the funnel plots corresponding to HD (Figure 6) and PD (Figure 7), studies are symmetrically distributed around the null effect line. This pattern suggests the absence of a clear bias, indicating that the included studies adequately represent the variability of observed effects in these modalities. Similarly, in the funnel plot for KT (Figure 8), most studies are evenly distributed on both sides of the null effect line, without a noticeable concentration in one direction. This suggests a low probability of overestimating the effects of KT on depression and indicates that available studies capture a balanced representation of the potential psychological benefits of transplantation.

Furthermore, the combined funnel plot (Figure 9) displays a balanced distribution of effects, with no apparent gaps in the lower part of the plot. This suggests no systematic omission of studies with negative or non-significant results. This is particularly relevant as the absence of studies reporting smaller or adverse effects could introduce a bias that overestimates the positive impact of KT.

To further substantiate these observations, Egger’s test was conducted, yielding a p-value of 0.42. This result confirms the absence of significant asymmetry in the distribution of studies, reinforcing that publication bias is unlikely. Consequently, these findings enhance the robustness of the meta-analysis, ensuring that the relationship between RRT and depression prevalence is accurately represented without the influence of systematic bias.

### 3.6. Sensitivity Testing: Depression by Renal Replacement Therapy Modality

The results indicate that the pooled SMD without each individual study remains stable, with values ranging between −1.06 and −0.69. The variation observed in the confidence intervals (95% CI) also remains within expected ranges, without affecting the statistical significance of the results. 

The highest variability in SMD was observed when excluding the study by Brito et al. [5] SMD = −0.69, 95% CI −0.98 to −0.40), suggesting that this study contributes moderately to the overall estimate. However, the exclusion of no studies resulted in significant changes in SMD, indicating high stability and robustness in the findings of the meta-analysis. 

The sensitivity test confirms that the results of the meta-analysis are robust and not overly dependent on any particular study.

### 3.7. Subgroup Analysis: Impact of Mean Age, Duration of Dialysis, and Comorbidities on Depression by Renal Replacement Therapy Modality

The results obtained from the meta-regression to evaluate the relationship between mean age, time on dialysis, and the presence of comorbidities (DM and HTN) with the prevalence of depression in patients undergoing RRT—including HD, PD, and KT—were based on data collected from the primary studies and are presented in Table 5.

Among HD patients, the 95% confidence interval (CI) for depression prevalence in older adults ranged from 48.1% to 66.3%. The regression analysis showed a β coefficient of −0.49 (*p* > 0.05), indicating no statistically significant association between age and depression prevalence in this group. In PD patients, the corresponding CI was between 44.5% and 60.1%, with no statistically significant association reported. For KT patients, depression prevalence remained between 39.8% and 50.7% across all age subgroups, and no statistically significant relationship was observed between age and depression prevalence.

Regarding dialysis duration, patients with more than 60 months on dialysis showed a depression prevalence ranging from 26.4% to 92.7% (95% CI), while those with a shorter duration had prevalence rates between 22.1% and 38.4% (95% CI). However, no statistically significant association between dialysis duration and depression prevalence was found.

In terms of comorbidities, HD and PD patients with DM or HTN exhibited a depression prevalence ranging from 30.1% to 62.3% (95% CI). Among KT patients, the prevalence within subgroups with these comorbidities was lower, ranging from 17.9% to 32.1% (95% CI), and no statistically significant differences were identified. Additionally, the original studies did not consistently specify whether patients had DM, HTN, or both, which limits our ability to analyze the independent effect of each comorbidity.

### 3.8. Meta-Regression: Influence of Age and Time of Dialysis on Depression in RRT Patients

A meta-regression analysis using a fixed-effects model was conducted to examine the influence of demographic variables on depression prevalence among patients undergoing RRT. The variables included were mean age in HD patients and time on dialysis (combining HD and PD patients).

The analysis revealed a β coefficient of −0.49 for age in HD patients, with a 95% confidence interval ranging from −1.53 to 0.54 (*p* = 0.32). For dialysis duration, the β coefficient was 0.15, with a 95% confidence interval from −0.20 to 0.50 (*p* = 0.36).

Regarding comorbidities, the β coefficient for DM was 1.84 (95% CI: −2.89 to 6.58), and for HTN, it was 1.18 (95% CI: −0.66 to 3.03), both with *p*-values greater than 0.05.

### 3.9. Distribution of Patients and Prevalence of Depression According to the Depression Measurement Instruments

Table 6 presents the prevalence of depression among patients with chronic kidney disease, categorized by treatment modality (hemodialysis, peritoneal dialysis, or kidney transplantation) and by assessment instrument (BDI-II, CES-D, and HADS). The reported prevalence rates are accompanied by their respective 95% confidence intervals, allowing for an overview of variations according to treatment type and evaluation method. 

### 3.10. Assessment of Heterogeneity and Robustness of Meta-Analysis by Type of Measurement Instruments

In all cases, HD and PD patients had a significantly higher prevalence of depression than transplant patients. No significant differences were observed between HD and PD on any of the instrument’s measurements. These trends were consistent regardless of the type of instruments used, supporting the robustness of the findings (Table 7). 

### 3.11. Subgroup Analysis by Age, Duration of Dialysis, and Comorbidities by Type of Measurement Instruments

The results concerning the prevalence of depression were analyzed according to patient age, duration of dialysis treatment, and the presence of comorbidities, using three different assessment instruments (BDI-II, CES-D, and HADS) across the treatment modalities examined (hemodialysis, peritoneal dialysis, and kidney transplantation). A detailed presentation of these results, including prevalence rates and 95% confidence intervals, is provided in Table 8. 

## 4. Discussion

This meta-analysis confirms that KT recipients exhibit significantly lower depression prevalence compared to patients undergoing HD or PD. This finding remained consistent across subgroups and assessment tools, with no signs of heterogeneity or publication bias, thus reinforcing its robustness. Although older age, prolonged dialysis, and comorbidities such as DM and HTN were associated with greater depressive symptoms, these associations did not reach statistical significance.

The lower prevalence of depression among KT recipients, regardless of the instrument used (BDI-II, HADS, and CES-D), supports the hypothesis that differences in emotional burden are primarily related to the type of RRT rather than the method of assessment. No significant differences were found between HD and PD patients, suggesting a similar psychological impact between these modalities.

These findings align with previous evidence that KT improves quality of life and emotional well-being [5,21,29,30] Reduced dependency on dialysis, fewer hospitalizations, and a more optimistic health perception have been proposed as factors contributing to this benefit. Transplant recipients consistently report higher scores across multiple quality-of-life domains, underscoring KT’s role as a psychological protective factor in CKD [22,28,30].

While HD and PD both impose significant psychological burdens, the absence of statistically significant differences between them may stem from shared challenges, such as physical fatigue, restricted autonomy, and social limitations. Studies by Khan et al. [31] and Yan et al. [32] have reported elevated depression rates in HD, while more regionally focused studies like those by Alencar et al. [33], Martínez et al. [29], and Molina et al. [34] observed more moderate figures. These inconsistencies are likely attributable to methodological differences, including sample size, single-center versus multicenter designs, and sociocultural context. Mosleh et al. [35] and Niebla et al. [36] highlighted how cultural stigma and data collection methods can influence the expression and reporting of depressive symptoms.

Prolonged dialysis duration and the presence of DM have been identified as relevant risk factors for depression in HD populations [31,37]. Similarly, older patients tended to show more moderate depressive symptoms, though causality cannot be inferred. In PD, studies such as Yang et al. [37] and Bazazzadeh et al. [38] a reported higher prevalence of depression, with educational level and comorbidities playing a significant role. KT again emerged as the modality with the most favorable psychological profile. Multiple investigations [30,39,40] confirmed lower depression levels among transplant recipients, especially when compared with patients on transplant waiting lists, who experience higher anxiety and psychological distress due to uncertainty [41].

Although PD showed a slight trend toward lower emotional burden compared to HD, the difference was not statistically significant. This reinforces KT as the option with the most consistently favorable psychological outcomes. These results suggest a clinically meaningful relationship rather than one due to chance.

Shared psychosocial stressors, functional limitations, and the inherent emotional weight of treatment likely contribute to the similarity in depression levels between HD and PD [37] Conversely, KT is associated with improved emotional regulation, which may be due to greater autonomy and reduced medical dependence. These interpretations are supported by Sandwijk et al. [28], Gurkan et al. [22], and Rubio et al. [27], who emphasized KT’s impact on emotional stability, social reintegration, and perceived health status.

The prevalence of depression found in this meta-analysis is consistent with intermediate values in the literature. While higher than those reported by Brito et al. [5], it aligns with findings from Kang et al. [26]. Other studies by Alavi et al. [21], Baykan et al. [7], Griva et al. [6], Sandwijk et al. [28], and Shdaifat et al. [13] corroborate the lower depressive burden observed in KT recipients across various populations.

Despite some heterogeneity in the study design and sample characteristics, the consistent trend toward better psychological outcomes in KT recipients enhances the validity of this meta-analysis. By integrating data from diverse cultural and regional contexts, this study offers a more generalizable and reliable estimate of depression prevalence in RRT populations.

Importantly, these findings were stable across depression assessment tools. While prevalence rates varied slightly depending on the instrument, the overall trend of lower depression among transplant recipients remained unchanged. This consistency strengthens the inference that RRT modality, not measurement method, drives the observed differences.

The literature also warns about the potential overestimation of depression in medically complex patients due to somatic symptom inclusion. Velescu et al. [42] and Takeuchi et al. [43] reported that fatigue and sleep disturbances can inflate depression scores, particularly in instruments not designed to separate somatic from emotional symptoms. In this context, HADS, developed to minimize somatic bias, may provide a more accurate reflection of psychological distress in CKD patients.

Our results reaffirm that dialysis modality alone does not determine depression risk, as HD and PD showed no significant differences. Instead, clinical and contextual factors—such as age, comorbidities, social support, and healthcare access—must be considered when designing psychological interventions for this population.

Although age, dialysis duration, and comorbidities did not show statistical significance, trends indicated that DM and prolonged dialysis duration were associated with greater depression risk, especially in HD and PD [35,37,44]. HTN, despite being highly prevalent in CKD, was not significantly associated with depression [31,38]. Nonetheless, its interaction with other conditions warrants further investigation.

Among transplant recipients, these associations were even weaker. Despite the high prevalence of HTN in this group, studies by Khan et al. [31] and Bazazzadeh et al. [38] did not find significant correlations with depressive symptoms. This suggests that treatment-related factors—particularly modality and duration—may be more influential than HTN itself [5,29].

Some studies also indicate greater vulnerability to depression among older dialysis patients. Al-Ali et al. [45] and Khan et al. [31] noted age-related increases in depressive symptoms among PD and HD patients, respectively. However, these findings should be interpreted with caution due to limitations in sample size and design. Notably, among KT recipients, age was not significantly associated with depression, reinforcing the hypothesis that KT serves as a psychological protective factor [22,27,46].

Ensuring equitable access to KT remains critical. Structural and administrative barriers can delay or prevent transplantation, increasing psychological distress among patients on waiting lists [7,13]. Moreover, psychosocial support can amplify the emotional benefits of KT. Studies by Sarhan et al. [30] and Chen et al. [39] found that interventions targeting mental well-being improve health perception and reduce depressive symptoms. Similarly, Gurkan et al. [22] and Rubio et al. [27] linked post-transplant emotional stability to better lifestyle adjustment and social reintegration.

Integrating mental health into CKD care is essential. Psychosocial strategies must be tailored to each RRT modality, with routine emotional assessments implemented both before and after transplantation to facilitate early detection of depressive symptoms.

Structured psychological interventions—including cognitive behavioral therapy, peer support, and counseling—have demonstrated significant benefits [47]. Special attention is needed for PD patients, whose home-based treatment limits contact with healthcare providers. Personalized support, caregiver education, and remote psychosocial services are critical. Gómez-Ibáñez et al. [48] highlighted the value of strengthening family support systems to protect the emotional health of this vulnerable population.

### 4.1. Clinical Implications and Psychological Strategies

Depression in CKD has a profound impact on patients’ quality of life and adherence to renal replacement therapy (RRT), particularly in HD populations. This burden has driven the development of psychological interventions with proven benefits for mental health. Cognitive behavioral therapy, counseling, and support groups have effectively reduced depressive and anxiety symptoms, highlighting the role of social and peer support networks [29,47,49,50,51]. Complementary approaches—including mindfulness, laughter therapy, and relaxation techniques—further support emotional well-being within multidisciplinary care models [52,53,54].

Untreated depression correlates with poor adherence, increased somatic symptoms, and higher mortality, underlining the need for routine psychological screening and timely intervention in dialysis units [35]. Khan et al. [31] showed that centers offering emotional support services report less progression of depressive symptoms over time. The integration of mental health professionals—psychologists, psychiatrists, and social workers—within RRT care teams enhances patients’ emotional adjustment and clinical outcomes. In the pre-transplant setting, early psychological intervention is essential as depression can hinder eligibility assessments [55]. Additionally, intradialytic exercise has demonstrated both functional and emotional benefits [56,57].

Home-based RRT modalities, including home hemodialysis and PD, offer potential advantages for emotional well-being. Findings presented by the Nephrology Department of Hospital Lucus Augusti de Lugo at the S.E.N. 2024 and IX Ibero-American Congress indicate that patients on home modalities experience less distress, aligning with D’Alessandri-Silva et al. [58], who reported sustained mood improvements after 12 months of home treatment. Nonetheless, despite similar depression prevalence between HD and PD, tailored psychological interventions for PD remain limited. Gómez-Ibáñez et al. [48] emphasize the need for customized strategies in PD, including caregiver education, stress management, and reinforcement of domestic support systems.

### 4.2. Perspectives and Future Directions

A priority for future research is evaluating the applicability of effective HD interventions—such as CBT, mindfulness, and peer support—for PD patients. Given PD’s home-based nature and reduced clinical contact, it is critical to develop strategies adapted to its unique context. Patients on PD manage their treatment largely independently and often face distinct emotional and logistical challenges. Therefore, support models must consider individual psychological needs and the family dynamics inherent to home care.

Home hemodialysis also appears to support psychological well-being, possibly due to its greater flexibility and autonomy. While current evidence remains limited, these factors may reduce the emotional toll associated with RRT. Further studies are needed to confirm these effects and guide context-specific mental health interventions.

Caregivers play a pivotal role in PD care, and their emotional well-being directly impacts patient outcomes. Structured programs targeting caregivers—including psychoeducation and stress management—could enhance emotional health for both patients and their families. Despite progress in HD and KT populations, PD remains underrepresented in psychological care research. Addressing this gap through targeted, personalized strategies is essential for delivering equitable mental health support across all RRT modalities.

### 4.3. Strengths and Limitations

This meta-analysis synthesizes data from 16 studies and over 26,301 patients, providing strong statistical power and generalizability. Low heterogeneity (I^2^ = 0%) and consistency across validated depression scales (BDI-II, CES-D, HADS) strengthen the validity of the findings. The results affirm that KT is associated with lower depression prevalence than dialysis, with no significant difference between HD and PD. The absence of publication bias (Egger’s test, *p* = 0.42) further supports the reliability of the analysis.

However, several limitations should be acknowledged. Some studies lacked detailed data on age, duration of dialysis, or comorbidities, limiting the precision of subgroup estimates. In HD and PD populations, DM and HTN were associated with higher depression rates, though the studies did not clarify if these conditions co-occurred, resulting in separate prevalence estimates. Additionally, few studies on KT provided data on these comorbidities—only four addressed DM and two reported on HTN—restricting interpretation.

Moreover, the strict inclusion criteria, which required studies to provide direct comparative data across HD, PD, and KT within the same analysis, substantially limited the number of eligible studies. While this approach enhanced the consistency and internal validity of the comparisons, it may have reduced the breadth and representativeness of the available literature.

Furthermore, due to insufficient reporting in the original studies, it was not possible to apply adjusted multivariable models to control for potential confounding factors such as age, comorbidities, or duration of dialysis. This limits the ability to draw causal inferences from the observed associations.

Although no publication bias was detected based on Egger’s test (*p* = 0.42), the limited number of studies per comparison may reduce the sensitivity of this method, and thus minor biases cannot be entirely excluded.

Study quality was assessed using the Newcastle–Ottawa Scale (NOS), a widely accepted tool for evaluating observational studies. However, the exclusive use of this instrument may have limited the evaluation of other dimensions of bias, such as reporting or selection nuances not captured by NOS.

Additionally, although this study reports on the prevalence of depression, prevalence rates alone may not fully reflect the overall psychological well-being of patients. External factors such as access to care, cultural context, and support systems may influence mental health outcomes and should be considered when interpreting these findings.

While the HADS has demonstrated validity in patients with CKD and was selected for its exclusion of somatic symptoms, its comparability with instruments such as the BDI-II and CES-D may vary. This should be considered when interpreting depression prevalence across studies using different tools.

Although the analysis focused on diabetes and hypertension due to their high prevalence and data availability, other potentially relevant comorbidities—such as anemia or cardiovascular conditions—were not examined. This narrows the clinical scope of the comorbidity analysis.

Lastly, although the search strategy was designed to identify studies reporting on depression specifically, it is possible that some literature addressing broader psychosocial dimensions may not have been captured. This should be considered when interpreting the comprehensiveness of the clinical recommendations.

Finally, while this meta-analysis provides important comparative evidence across RRT modalities, further experimental or mechanistic studies are needed to explore the causal pathways linking depression and renal replacement therapy.

## 5. Conclusions

This meta-analysis confirms that depression is highly prevalent in patients with CKD undergoing renal RRT. However, the magnitude of this psychological burden varies depending on the treatment modality, being higher in dialysis patients and significantly lower in those who have received a kidney transplant.

The findings suggest that KT not only improves renal function and survival but could also have a positive impact on a patient’s mental health, significantly reducing depressive symptoms. This indicates that the psychological burden in dialysis patients may not depend solely on the type of treatment but on additional factors that require further exploration.

In conclusion, this study highlights the lower prevalence of depression in transplant patients compared to those undergoing dialysis, suggesting the importance of improving access to KT and strengthening psychological support for dialysis patients. While no significant associations were found between depression and factors such as comorbidities or dialysis duration, the observed trends warrant further research. Future studies should explore psychosocial factors—particularly in peritoneal dialysis patients—such as social support, autonomy, and emotional coping, to inform more personalized mental health strategies across RRT modalities.

Future research should focus on adapting and evaluating psychological interventions—such as cognitive behavioral therapy, mindfulness, and structured peer support—according to the type of renal RRT. In the specific case of patients undergoing peritoneal dialysis, incorporating caregiver support would also be relevant to address the unique psychosocial needs associated with this home-based modality.

## Figures and Tables

**Table 1:** Assessment of methodological quality of included studies using the Newcastle–Ottawa Scale (NOS).

Study	Selection (0–4)	Comparability (0–2)	Outcomes (0–3)	Total Score (0–9)	Quality
Alavi M.N. et al., 2009 [21]	3	2	3	8	High
Baykan H. et al., 2012 [7]	3	1	2	6	Moderate
Brito D.C. et al., 2019 [5]	4	2	3	9	High
Brown E.A. et al., 2010 [8]	3	1	2	6	Moderate
Griva K. et al., 2014 [6]	4	2	3	9	High
Gurkan A. et al., 2015 [22]	3	1	2	6	Moderate
Hiramatsu T. et al., 2020 [23]	3	1	2	6	Moderate
Iyasere O.U. et al., 2016 [10]	4	2	3	9	High
Jung H.Y. et al., 2019 [24]	4	2	3	9	High
Kalender B. et al., 2007 [25]	3	2	3	8	High
Kang M.S. et al., 2024 [26]	4	2	3	9	High
Kovacs A.Z. et al., 2011 [3]	4	1	3	8	High
Rubio R. et al., 2012 [27]	3	1	2	6	Moderate
Sandwijk M.S. et al., 2019 [28]	3	1	2	6	Moderate
Shdaifat E.A. et al., 2022 [13]	3	2	2	7	Moderate
Turkmen K. et al., 2012 [4]	3	1	2	6	Moderate

Methodological quality assessment of included studies using the Newcastle–Ottawa Scale (NOS). The scale evaluates three domains: selection (0–4 points), comparability (0–2 points), and outcomes (0–3 points), with a total score ranging from 0 to 9. Studies are classified as high quality (7–9 points), moderate quality (5–6 points), or low quality (0–4 points).

**Table 2:** Characteristics of the studies included in the meta-analysis and depression measurement instruments.

Study	Country	Sample Size (*n*)	Groups Studied	Depression (%)	Type of Instruments Used
Alavi M.N. et al., 2009 [21]	Iran	*n* = 163	HD = 63 KT = 100	HD = 65.3 KT = 39	BDI-II
Baykan H. et al., 2012 [7]	Turkey	*n* = 123	HD = 41 PD = 41 HC = 41	HD = 33.3 PD = 29.3 HC = 14.6	BDI-II
Brito D.C. et al., 2019 [5]	Brazil	*n* = 205	DL = 130 KT = 75	DL (HD + PD) = 41.7 KT = 32.3	CES-D
Brown E.A. et al., 2010 [8]	United Kingdom	*n* = 140	HD = 70 PD = 70	HD = 26 PD = 10	HADS
Griva K. et al., 2014 [6]	Singapore	*n* = 433	HD = 232 PD = 201	HD = 8.07 PD = 9.00	HADS
Gurkan A. et al., 2015 [22]	Turkey	*n* = 214	HD = 138 KT = 76	HD = 58.9 KT = 25	BDI-II
Hiramatsu T. et al., 2020 [23]	Japan	*n* = 65	HD = 38 PD = 27	HD = 39.3 PD = 39.3	HADS
Iyasere O.U. et al., 2016 [10]	United Kingdom	*n* = 251	HD = 122 PD = 129	HD = 23.8 PD = 38.8	HADS
Jung H.Y. et al., 2019 [24]	South Korea	*n* = 989	HD = 652 PD = 337	HD = 39.5 PD = 58.1	CES-D
Kalender B. et al., 2007 [25]	Turkey	*n* = 181	HD = 68 PD = 47 HC = 66	HD = 33.8 PD = 12.8 HC = 19.2	BDI-II
Kang M.S. et al., 2024 [26]	South Korea	*n* = 21.809	HD = 15.537 PD = 2.112 KT = 4.160	Dialysis (HD + PD) = 22.8 KT = 8.6	CES-D
Kovacs A.Z. et al., 2011 [3]	Hungary	*n* = 1.075	DL = 187 KT = 888	DL (HD + PD) = 39.4 KT = 19.5	CES-D
Rubio R. et al., 2012 [27]	Spain	*n* = 119	HD = 74 PD = 45	HD = 28.4 PD = 44.4	BDI-II
Sandwijk M.S. et al., 2019 [28]	Netherlands	*n* = 163	HD = 30 KT = 30 CT = 30 Ca Remission = 30 HC= 43	HD = 33.3 KT = 16.7 CT = 30.0 Ca Remission = 23.3 HC = 12.1	HADS
Shdaifat E.A. et al., 2022 [13]	Jordan	*n* = 217	HD = 105 PD = 20 KT = 92	HD = 39.4 PD = 39.4 KT = 19.5	BDI-II
Turkmen K. et al., 2012 [4]	Turkey	*n* = 154	HD = 90 PD = 64	HD = 23.3 PD = 65.6	BDI-II

BDI-II (Beck Depression Inventory-Second Edition): Cronbach’s α > 0.80; Ca Remission (Cancer in Remission); CES-D (Center for Epidemiological Studies Depression Scale): Cronbach’s α > 0.85; CT (Chemotherapy); DL (Dialysis); HADS (Hospital Anxiety and Depression Scale ): Cronbach’s α > 80; HC (Healthy Control Group); HD (Hemodialysis); KT (Transplant Kidney); n: Total number of patients in the studied sample; PD (Peritoneal Dialysis).

**Table 3:** Prevalence of depression by type of RRT.

Group Prevalence	Depression (%)	Standard Error (%)
HD	35.56	1.06
PD	35.09	1.05
RT	25.33	0.96

HD (Hemodialysis); PD (Peritoneal Dialysis); KT (Renal Transplant).

**Table 4:** Standardized Mean Difference (SMD) and 95% Confidence Intervals (CIs) for depression prevalence in RRT modalities.

Group	SMD	95% CI (Lower)	95% CI (Upper)
HD	−0.66	−1.01	−0.30
PD	−0.76	−1.25	−0.26
KT	−1.12	−1.35	−0.89

HD (Hemodialysis); KT (Kidney Transplantation); PD (Peritoneal Dialysis); Standardized mean difference (SMD).

**Table 5:** Mean age, duration of dialysis, and comorbidities.

Study	Mean Age (Years ± SD)	Duration of Dialysis (Months ± SD)/Months [IQR]	Diabetes (%)	Hypertension (%)
Alavi M.N. et al., 2009 [21]	HD = 55.30 ± 14.50 KT = 40.60 ± 14.64	HD = 36.80 ± 17.6 KT = 59.50 ± 28.00	HD = 26.00 KT = 20.00	HD = 65.00 KT = 35.00
Baykan H. et al., 2012 [7]	HD = 49.09 ± 12.00 PD = 40.63 ± 14.09 KT = 42.00 ± 11.60	-	-	-
Brito D.C. et al., 2019 [5]	HD = 54.50 ± 12.70 KT = 13,251.90 ± 11.30	HD = 120.10 ± 8.10 KT = 44.5 0 ± 39.60	**-**	-
Brown E.A. et al., 2010 [8]	HD = 73.40 ± 5.10 PD = 73.10 ± 5.50	HD = 31.40 ± 26.50 PD = 30.50 ± 28.30	HD = 32.00 PD = 13.00	-
Griva K. et al., 2014 [6]	HD = 58.92 ± 12.59 KT = 53.52 ± 10.47	HD = 42.12 ± 38.76 KT = 96.36 ± 60.96	HD = 41.70 KT = 37.30	HD = 18.90 KT = 21.90
Gurkan A. et al., 2015 [22]	HD = 54.70 ± 12.50 KT = 38.40 ± 11.90	HD = 60.44 ± 42.50 KT = 7.84 ± 1.87	-	-
Hiramatsu T. et al., 2020 [23]	HD = 66.80 ± 8.40 PD = 63.10 ± 11.00	-	HD = 26.00 PD = 20.00	HD = 65.00 PD = 35.00
Iyasere O.U. et al., 2016 [10]	HD = 75.00 ± 11.00 PD =76.00 ± 11.00	HD = 27.50 [15.5–39.5] PD = 22.00 [11,12,13,14,15,16,17,18,19,20,21,22,23,24,25,26,27,28,29,30,31,32,33,34,35]	HD = 50.50 PD = 49.50	HD = 5.90 PD = 8.90
Jung H.Y. et al., 2019 [24]	HD = 56.60 ± 13.50 PD = 51.60 ± 12.80	-	HD = 62.40 PD = 49.00	-
Kalender B. et al., 2007 [25]	HD = 51.00 ± 15.20 PD = 48.70 ± 16.50	HD = 30.60 ± 72.90 PD = 24.40 ± 23.30	-	-
Kang M.S. et al., 2024 [26]	DL = 58.32 ± 14.80 KT = 47.43 ± 12.87	-	DL = 86.70 KT = 84.70	DL = 98.40 KT = 97.70
Kovacs A.Z. et al., 2011 [3]	HD = 49.00 ± 12.00 KT = 49.00 ± 13.00	HD = 45.20 ± 34.07 KT = 45.20 ± 34.00	HD = 18.00 KT = 17.00	-
Rubio R. et al., 2012 [27]	n = 58.80 ± 14.20	-	-	-
Sandwijk M.S. et al., 2019 [28]	HD = 57.60 ± 13.63 KT = 56.60 ± 17.63	-	-	-
Shdaifat E.A. et al., 2022 [13]	HD = 46.91 ± 15.77 PD = 46.15 ± 15.29 KT = 33.15 ± 10.61	HD = 80.38 ± 84.18 PD = 92.70 ± 96.78 KT = 105.70 ± 71.97	HD = 13.30 PD = 19.50 KT = 0.00	-
Turkmen K. et al., 2012 [4]	HD = 55.00 ± 15.70 PD = 52.40 ± 15.30	HD = 22.7 0 ± 13.10 PD = 19.80 ± 14.30	HD = 30.00 PD = 34.40	HD = 21.10 PD = 14.10

DL (Dialysis); HD (Hemodialysis); PD (Peritoneal Dialysis); KT (Kidney Transplantation).

**Table 6:** Distribution of patients and prevalence of depression according to treatment modality and depression measurement instrument.

Meta-Analysis by Instruments Type		Comparison Between Treatment Modality
BDI-II:	HD: 42.30% (IC 95%: 35.10–49.50). PD: 38.70% (IC 95%: 30.20–47.20). KT: 28.40% (IC 95%: 20.50–36.30).	HD > PD > KT
CES-D:	HD: 37.50% (IC 95%: 32.10–42.90). PD: 35.80% (IC 95%: 29.40–42.2). KT: 20.1% (IC 95%: 15.3–24.9).	HD > PD > KT
HADS:	HD: 25.60% (IC 95%: 18.30–32.90). PD: 24.30% (IC 95%: 16.80–31.80). KT: 16.70% (IC 95%: 10.50–22.90).	HD > PD > KT

BDI-II (Beck Depression Inventory-Second Edition); CES-D (Center for Epidemiologic Studies Depression Scale); HADS (Hospital Anxiety and Depression Scale); HD (Hemodialysis); KT (Kidney Transplantation); PD (Peritoneal Dialysis).

**Table 7:** Meta-analysis of depression prevalence by measurement instrument and treatment modality.

Category	BDI-II	CES-D	HADS
I^2^	85%	72%	89%
Cochran’s Q (*p*-value)	45.32, *p* < 0.001	32.15, *p* < 0.001	50.45, *p* < 0.001
Combined Prevalence	42.30% (95% CI: 35.10–49.50)	37.50% (95% CI: 32.10–42.9)	25.60% (95% CI: 18.30–32.90)
Egger’s Test	*p* = 0.12	*p* = 0.18 (not significant)	*p* = 0.22 (not significant)
Funnel Plot	No evident asymmetry	No evident asymmetry	No evident asymmetry
Sensitivity Range	[40.10–44.50%]	[35.80–39.20%]	[23.50–27.80%]
Subgroup: Advanced Age	Greater prevalence in HD and PD	Lower prevalence in HD and PD	Greater prevalence in HD and PD
Subgroup: Duration of Dialysis (≥60 months)	Greater prevalence	Lower prevalence	Greater prevalence
Subgroup: Comorbidities (DM & HTN)	Greater prevalence in patients with MD and HTN	Variation observed in patients with MD and HTN	Greater prevalence in patients with MD and HTN
Meta-regression: Age	β = −0.45 (95% CI: −1.40 to 0.50), *p* = 0.35	β = −0.50 (95% CI: −1.55 to 0.55), *p* = 0.34	β = −0.40 (95% CI: −1.30 to 0.50), *p* = 0.38
Meta-regression: Duration of Dialysis	β = 0.18 (95% CI: −0.15 to 0.51), *p* = 0.28	β = 0.20 (95% CI: −0.10 to 0.50), *p* = 0.19	β = 0.15 (95% CI: −0.20 to 0.50), *p* = 0.40

BDI-II (Beck Depression Inventory-Second Edition); CES-D (Center for Epidemiologic Studies Depression Scale); DM (Diabetes Mellitus); HADS (Hospital Anxiety and Depression Scale); HD (Hemodialysis); HTN (Hypertension); I^2^ (Heterogeneity Index); PD (Peritoneal Dialysis); *p* (*p*-value).

**Table 8:** Subgroup analysis of depression prevalence by measurement instrument and treatment modality.

Measurement Instrument	Age	Duration of Dialysis	Comorbidities
BDI-II	HD: Older patients (>60 years) had a depression prevalence of 48.50% (95% CI: 40.20–56.80). PD: Older patients (>60 years) had a depression prevalence of 42.3% (95% CI: 34.10–50.50). KT: No significant association was observed between older age and depression (prevalence = 28.40%, 95% CI: 20.50–36.30).	HD: Patients with over 60 months on dialysis showed a depression prevalence of 52.10% (95% CI: 44.30–59.90). PD: Patients with over 60 months on dialysis showed a depression prevalence of 47.80% (95% CI: 39.20–56.40). KT: No significant association was observed between duration of dialysis and depression.	HD: Patients with MD and HTN had a depression prevalence of 55.30% (95% CI: 47.1–63.5). PD: Patients with MD and HTN had a depression prevalence of 50.2% (95% CI: 41.80–58.60). KT: No significant association was observed between comorbidities and depression.
CES-D	HD: Older patients (>60 years) had a depression prevalence of 42.50% (95% CI: 36.10–48.90). PD: Older patients (>60 years) had a depression prevalence of 38.7% (95% CI: 32.30–45.10). KT: No significant association was observed between older age and depression (prevalence = 20.10%, 95% CI: 15.30–24.90).	HD: Patients with over 60 months on dialysis showed a depression prevalence of 47.30% (95% CI: 40.50–54.10). PD: Patients with over 60 months on dialysis showed a depression prevalence of 43.20% (95% CI: 36.40–50.00). KT: No significant association was observed between duration of dialysis and depression.	HD: Patients with MD and HTN had a depression prevalence of 50.80% (95% CI: 43.20–58.40). PD: Patients with MD and HTN had a depression prevalence of 45.60% (95% CI: 38.00–53.20). KT: No significant association was observed between comorbidities and depression.
HADS	HD: Older patients (>60 years) had a depression prevalence of 30.20% (95% CI: 22.50–37.90). PD: Older patients (>60 years) had a depression prevalence of 28.70% (95% CI: 20.80–36.60). KT: No significant association was observed between older age and depression (prevalence = 16.70%, 95% CI: 10.50–22.90).	HD: Patients with over 60 months on dialysis showed a depression prevalence of 35.4% (95% CI: 27.30–43.5). PD: Patients with over 60 months on dialysis showed a depression prevalence of 32.10% (95% CI: 24.0–40.2). KT: No significant association was observed between duration of dialysis and depression.	HD: Patients with MD and HTN had a depression prevalence of 38.5% (95% CI: 30.20–46.80). PD: Patients with MD and HTN had a depression prevalence of 34.20% (95% CI: 26.10–42.30). KT: No significant association was observed between comorbidities and depression.

BDI-II (Beck Depression Inventory-Second Edition); CES-D (Center for Epidemiologic Studies Depression Scale); DM (Diabetes Mellitus); (HADS (Hospital Anxiety and Depression Scale); HD (Hemodialysis); HTN (Hypertension); KT (Kidney Transplantation); PD (Peritoneal Dialysis).

## Data Availability

No new data were created or analyzed in this study.

## Abbreviations

The following abbreviations are used in this manuscript:

BDI-II	BDI-II: Beck Depression Inventory-Second Edition
Ca Remission	Cancer in Remission
CES-D	Center for Epidemiologic Studies Depression Scale
CKD	Chronic Kidney Disease
CT	Chemotherapy
DL	Dialysis
DM	Diabetes Mellitus
HADS	Hospital Anxiety and Depression Scale
HC	Healthy Control Group
HD	Hemodialysis
HTN	Hypertension
I^2^	Heterogeneity index
KT	Kidney Transplantation
n	Total number of patients in the studied sample
*p*	*p*-value
PD	Peritoneal Dialysis
SMDs	Standardized mean differences
